# Fluid Responsiveness Is Associated with Successful Weaning after Liver Transplant Surgery

**DOI:** 10.3390/jpm14040429

**Published:** 2024-04-18

**Authors:** Ricardo Castro, Pablo Born, Felipe Muñoz, Camila Guzmán, Eduardo Kattan, Glenn Hernandez, Jan Bakker

**Affiliations:** 1 Departamento de Medicina Intensiva, Facultad de Medicina, Pontificia Universidad Católica de Chile, Santiago Centro, Santiago 8330074, RM, Chile; piborn@uc.cl (P.B.); fimunoz2@uc.cl (F.M.); cfguzman@miuandes.cl (C.G.); ejkattan@uc.cl (E.K.); glennguru@gmail.com (G.H.); jan.bakker@erasmusmc.cl (J.B.); 2 Hospital Clinico UC-CHRISTUS, Pontificia Universidad Católica de Chile, Santiago Centro, Santiago 8330024, RM, Chile; 3 Department of Intensive Care, Erasmus MC University Medical Center, P.O. Box 2040, 3000 Rotterdam, The Netherlands; 4 Department of Pulmonology and Critical Care, Columbia University Medical Center, New York, NY 10019, USA; 5 NYU School of Medicine, Langone, New York, NY 10016, USA

**Keywords:** fluid overload, fluid responsiveness, passive leg raising, spontaneous breathing trial

## Abstract

A positive fluid balance may evolve to fluid overload and associate with organ dysfunctions, weaning difficulties, and increased mortality in ICU patients. We explored whether individualized fluid management, assessing fluid responsiveness via a passive leg-raising maneuver (PLR) before a spontaneous breathing trial (SBT), is associated with less extubation failure in ventilated patients with a high fluid balance admitted to the ICU after liver transplantation (LT). We recruited 15 LT patients in 2023. Their postoperative fluid balance was +4476 {3697, 5722} mL. PLR maneuvers were conducted upon ICU admission (T1) and pre SBT (T2). Cardiac index (CI) changes were recorded before and after each SBT (T3). Seven patients were fluid-responsive at T1, and twelve were responsive at T2. No significant differences occurred in hemodynamic, respiratory, and perfusion parameters between the fluid-responsive and fluid-unresponsive patients at any time. Fluid-responsive patients at T1 and T2 increased their CI during SBT from 3.1 {2.8, 3.7} to 3.7 {3.4, 4.1} mL/min/m^2^ (*p* = 0.045). All fluid-responsive patients at T2 were extubated after the SBTs and consolidated extubation. Two out of three of the fluid-unresponsive patients experienced weaning difficulties. We concluded that fluid-responsive patients post LT may start weaning earlier and achieve successful extubation despite a high postoperative fluid balance. This highlights the profound impact of personalized assessments of cardiovascular state on critical surgical patients.

## 1. Introduction

Fluid balance, the net difference between intake and output, traditionally guides undifferentiated fluid management decisions in critically ill patients; however, fluid management in mechanically ventilated patients is complex. In general, a positive fluid balance is associated with weaning challenges and adverse outcomes such as extubation failure and higher mortality rates [1,2]. According to a recent study, a one-liter surplus on day 3 in the ICU can elevate the mortality risk by 19% [3]. Conversely, a negative fluid balance is associated with improved survival [1,4,5].

Nevertheless, even in the presence of fluid accumulation, some patients do not exhibit detrimental effects, which may be attributable to a condition known as fluid tolerance. This recently defined concept describes the degree to which a patient can tolerate fluid administration without causing organ dysfunction [6]. Thus, a positive fluid balance alone may not warrant reversal; its clinical impact is significant when associated with fluid overload, a condition that precedes organ dysfunction [5,7] and correlates with elevated morbidity and mortality [8,9,10,11]. Regrettably, most characterizations of fluid overload only define it arithmetically as a gain of 5–10% from admission weight without functional considerations [5,7]. Nevertheless, the pathophysiology of fluid overload is intricate and includes a net volume increase, redistribution of fluid from the peripheral to central veins, diminished fluid elimination due to renal impairment, and endothelial dysfunction [12], all of which may result in organ failure.

This complexity underscores the need for personalized diagnostic approaches that not only quantify fluid balance but also assess its physiological impact on individual cardiovascular function. The classic paradigm of fluid management, primarily guided by aggregate metrics and standardized protocols, often overlooks nuanced physiological variances among individuals. In this context, passive leg raising (PLR) appears to be a helpful non-invasive strategy for the assessment of fluid responsiveness to bridge the gap between the theoretical understanding of the consequences of fluid overload and the practical, personalized bedside decision making regarding volume management. PLR is a simple and reversible maneuver that mimics rapid fluid loading by shifting venous blood from the legs towards the intrathoracic compartment, increasing ventricular preloads, and thereby increasing stroke volume and cardiac output [13]. A positive PLR maneuver is a strong indicator of fluid responsiveness in mechanically ventilated patients [13,14], revealing a ventricular systolic function operating along the plateau phase of the Frank–Starling curve [15].

A negative PLR maneuver, indicating fluid unresponsiveness, prompts the safe removal of excess fluid [16]; similarly, when performed before a spontaneous breathing trial (SBT) in nonsurgical patients [17], it is predictive of weaning failure of cardiac origin [18,19]. During the weaning phase, the withdrawal of positive pressure increases venous return. Still, a state of fluid unresponsiveness can impede an appropriate increase in cardiac output [17,20,21], essential for matching the increased VO_2_ that occurs during the transition to spontaneous ventilation [20]. However, the predictive value of fluid responsiveness, assessed using PLR before weaning, has not yet been explored in surgical patients [17].

In the dynamic and critical setting of intensive care following liver transplant surgery, fluid balance management remains a cornerstone of patient care. These patients face critical fluid management challenges owing to their previously altered fluid homeostasis physiology [22] and surgery-induced fluid shifts, which increase the risk of post-transplant complications [22,23,24,25]. These facts make liver transplant patients a distinctively informative group for studying the predictive value of PLR, which could potentially guide more precise fluid therapy and improve the weaning process through fluid responsiveness determination.

This study aimed to explore whether fluid responsiveness, assessed using a PLR maneuver before a weaning trial, was associated with favorable ventilatory outcomes in a cohort of mechanically ventilated patients admitted to the ICU after liver transplantation, all of whom presented a high postoperative fluid balance. We postulated that a personalized approach to fluid management considering cardiovascular responses using PLR could expedite the weaning process in these patients. In addition, such an approach could be helpful in critical care practices for postoperative patients undergoing great abdominal surgery.

## 2. Materials and Methods

We conducted a prospective observational study on postoperative liver transplant patients at an academic tertiary care center ICU in 2023. This was an ancillary study using the FLOW protocol (NCT04496583 registration in clinicaltrials.gov), and patients were recruited after obtaining approval from the Institutional Review Board. The study was exempt from requiring informed consent owing to its observational nature, as approved by our Ethics Committee (ID 201015001-2021). This FLOW study was funded by FONDECYT, grant No 1200248-2020, from the Agencia Nacional de Investigación y Desarrollo (ANID), Chile.

### 2.1. Study Population

We recruited patients aged >18 years who were admitted to the ICU after liver transplantation for postoperative management. Five patients were living-donor liver transplant recipients. The exclusion criteria were acute postoperative, hemorrhagic, or vascular complications such as bleeding or hepatic artery or portal vein thrombosis that required additional surgeries that would result in the obligatory maintenance of mechanical ventilation. Patients were included when the research team was available (business days from 8:00 am to 12:00 pm). All patients had a central venous catheter and an arterial line fitted upon ICU entry.

Demographic and clinical profiles, along with standard ICU monitoring and fluid balance metrics, were systematically documented. In addition to the fluid balance, we determined the estimated plasma volume (ePV) using the Strauss-derived [26] and precision-adjusted Duarte formulas [27] and calculated the plasma volume status (PVS) using the Kaplan–Hakim formula [28]. The PVS indicates the actual versus ideal plasma volume disparity calculated and was determined after ICU admission and before the SBT for comparison with the fluid balance and responsiveness status. The PVS offers a percentage-based evaluation of plasma volume that correlates well with the plasma volume estimation when measured using a radio-labeled albumin assay [26,29]. We used the suggested cut-off of 6.3% for this parameter [30].

### 2.2. Bioreactance Monitoring

We used a non-invasive bioreactance monitor (Cheetah-Starling SV©, Baxter, Deerfield, IL, USA) because it provided continuous real-time data on cardiovascular function with an inexistent risk of complications and increased patient comfort, which is especially important in the postoperative setting. Bioreactance analyzes the relative phase shift of an oscillating current passing through the thoracic cavity [31]. The device automatically recorded all data every 8 s to an exportable spreadsheet file.

### 2.3. Study Procedures

#### 2.3.1. Hemodynamic Monitoring

After recruitment, a bioreactance-monitoring device was placed on each patient. We recorded the cardiac index (CI), stroke volume index (SVI), stroke volume variation (SVV), and thoracic fluid content (TFC) as the main hemodynamic variables. We assessed the absolute and relative positional variations in hemodynamic measures across the baseline and during passive leg raises at T1 and T2, as well as before and after an SBT, in addition to the monitor’s automated data output. The default 10% fluid responsiveness threshold of the device was utilized. A dual-investigator review of the patient charts ensured a detailed capture of the impact of the PLR maneuver on hemodynamic parameters.

#### 2.3.2. Spontaneous Breathing Trial

After the patient’s condition stabilized following liver transplant surgery, sedation was withdrawn, and the process of gradually reducing mechanical ventilation support was initiated to transition from controlled to spontaneous ventilation. A protocolized weaning program was implemented to prepare the patients for extubation, which involved assessing their hemodynamic stability, peripheral perfusion, and neurological function, including consciousness and cough reflex. Once the patients could tolerate a reduced applied airway pressure support of 10 cm H_2_O, an SBT was conducted for 30 min using a standardized protocol that included inspiratory pressure augmentation of 7 cm H_2_O and zero positive end-expiratory pressure. No SBTs were performed using the T-piece.

Upon successful completion of the SBT, the patient was extubated if deemed eligible by the attending physician. The patient was monitored for 48 h to ensure that reintubation was not required, and the maintenance of spontaneous ventilation by day 7 was considered consolidated extubation. A standardized post-extubation respiratory support protocol, including an oxygen mask, a high-flow nasal cannula (HFNC), and non-invasive ventilation, was available if needed. The respiratory therapy team managed the entire weaning process, which provided airway secretion clearance, bronchodilators, or other necessary interventions under physician supervision. Additionally, a post-extubation swallowing screening assessment was performed on all patients.

#### 2.3.3. Passive Leg Raising

**Figure 1:**
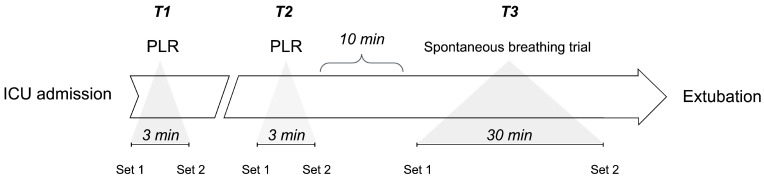
Timeline of three key cardiovascular monitoring time points in postoperative liver transplant patients since their ICU entry.

Stable data for the baseline SVI were obtained after 3 min in a semi-recumbent position at 45°. The first PLR maneuver (T1) was initiated by placing the patient in the supine position with the motorized ICU bed system and simultaneously raising their legs to 45° with the assistance of two operators. The legs were secured using a rigid-cushioned frame. The results were automatically displayed on the screen 3 min after starting the test. After 6 min, the patient was returned to the previous position. A second PLR maneuver was performed before the SBT, as described for the first maneuver (T2). Before each PLR maneuver, the patients were informed of the test to avoid stressful triggers that could hinder the results. In addition, the same hemodynamic parameters were recorded before and after the SBTs (T3), with at least a 10 min lapse after T2 (Figure 1). At all times, attending physicians were unaware of the fluid responsiveness state.

### 2.4. Statistical Methods

The study participants’ baseline demographic and general hemodynamic parameters are presented as the median and 25–75 interquartile ranges {IQR 25, 75} and proportions. Comparisons between fluid-responsive and fluid-unresponsive patients were performed using the Wilcoxon rank-sum test for continuous variables, owing to the nonparametric distribution of the data. Multiple linear regression analyses were performed to examine the influence of the MELD score, fluid responsiveness status, fluid balance, and CI on time to SBT and time to extubation. The significance level was set at *p* < 0.05. Data analyses and graphical representations were conducted using the DATAtab Online Statistics Calculator (DATAtab e.U., Graz, Austria, https://datatab.net accessed on 1 March 2024).

## 3. Results

**Figure 2:**
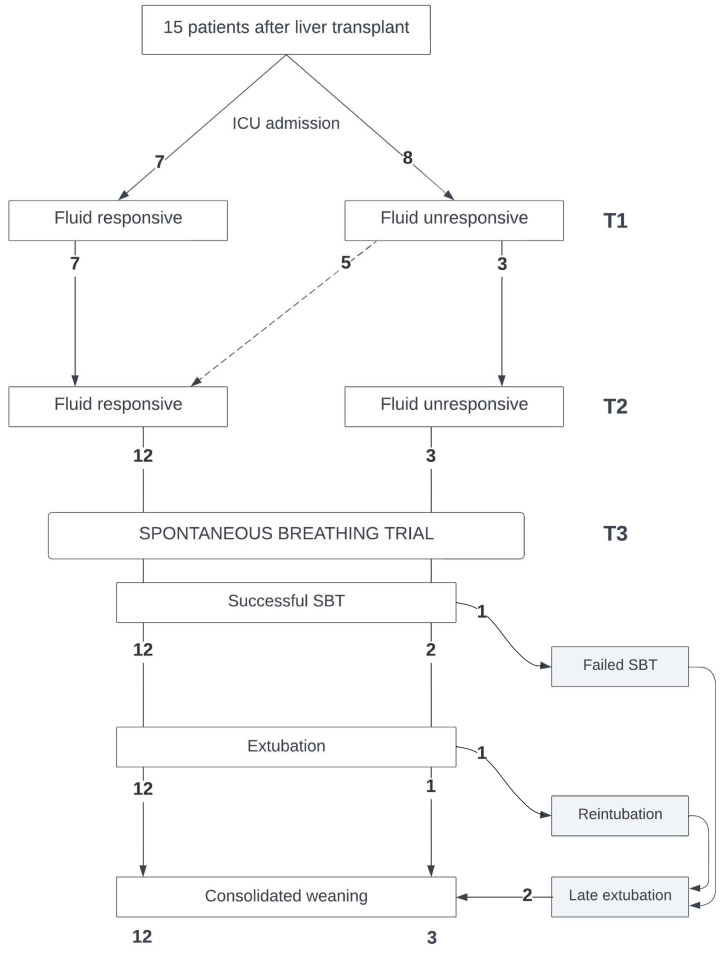
Flowchart describing the ventilatory outcomes of fifteen liver transplant patients after ICU admission, based on their fluid responsiveness and progression through a spontaneous breathing trial. Consolidated weaning was defined as no reintubation up to day 7 after extubation (*n* = number of patients).

Fifteen patients were recruited (general characteristics: Table 1; patient flow: Figure 2). Upon ICU admission, all patients presented with a weight increase (9.3 {8.4, 10.5} kg since hospital admission, 15% increase), high fluid balance (4480 {3698, 5723} mL), and high PVS (13 {8, 17} %).

**Figure 3:**
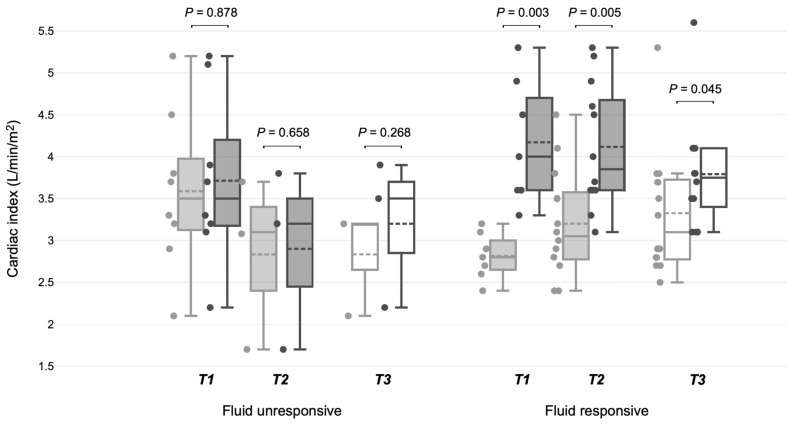
Cardiac index variations across different time points in fluid-responsive and fluid-unresponsive post-surgical liver transplant mechanically ventilated patients. T1: PLR maneuver upon ICU entry, T2: PLR before spontaneous breathing trial, T3: 30 min period between the start and conclusion of the spontaneous breathing trial.

Upon ICU admission, seven patients were fluid-responsive, whereas eight were fluid-unresponsive. At T2, of the eight initially fluid-unresponsive patients, five became fluid-responsive (Figure 2). General hemodynamic and respiratory parameters were similar in the fluid-responsive and fluid-unresponsive patients at T1 and T2, and maintained similar values during the SBTs (T3) (Table 2a). Heart rate was comparable between the fluid-responsive and fluid-unresponsive patients at T1 and T2, and continued to be alike during the SBTs (Table 2b). Fluid-responsive patients started the SBT earlier (at 14 {12, 27} h vs. 35.5 {20; 112} h; *p* = 0.06) and achieved successful extubation sooner (at 20 {15; 40} h vs. 45 {42; 121} h; *p* = 0.045) than their fluid-unresponsive counterparts. All fluid-responsive patients at T2 passed the SBT successfully and were extubated without complications. Conversely, two of the three fluid-unresponsive patients at T1 who remained in that state until their SBT had weaning problems: one failed the SBT, and the other had to be reintubated (Figure 2, Table S1). The SVI increased significantly in the fluid-responsive patients at T1 and T2, unlike in the fluid-unresponsive patients (Table 2b, Figure 3). During the SBTs, significant increases in the CI and SVI were observed in the patients previously identified as fluid-responsive at T1 and T2. There were no statistically significant changes in the CVP, SvO_2_, pCO_2_, dCO_2_, or TFC in any group at T1, T2, or T3.

Fluid balance and PVS values were similar between the fluid-responsive and fluid-unresponsive patients at T1 and T2 (Table 3a,b). Fluid balance and PVS were not associated with fluid responsiveness at T1 or T2 (Table 3a,b).

T1 corresponds to the first PLR maneuver, T2 corresponds to the second one (before the spontaneous breathing trial), and T3 corresponds to the 30 min period between the start and conclusion of the SBT. The set of hemodynamic parameters recorded for comparative analyses included the CI, SVI, SVV, CVP, and TFC (see main text for details).

In the case of PLR maneuvers, Set 1 corresponds to baseline parameters and Set 2 corresponds to the parameters at the end of the test. In the case of the SBT, Set 1 corresponds to the hemodynamic parameters recorded for comparative analyses, including the CI, SVI, SVV, CVP, and TFC (see main text for details), before starting the SBT and Set 2 corresponds to the recording of the same parameters at the end of the trial.

## 4. Discussion

The findings of our observational study, albeit small-scale, highlight the profound impact that personalized fluid management may have on post-liver-transplant recovery. Our data suggest that identifying the condition of fluid responsiveness after surgery is potentially beneficial for achieving earlier weaning and successful extubation, independent of fluid balance. Notably, we observed that a high fluid balance was not equivalent to a fluid-unresponsiveness state. Fluid responsiveness, assessed using PLR maneuvers, delineates a subgroup of patients who would significantly benefit from tailored fluid management strategies, manifesting in more successful weaning and extubation outcomes. This correlation not only proposes fluid responsiveness as a determinant of patient-specific care but also underscores the limitations of conventional, one-size-fits-all fluid management and removal protocols.

All patients who emerged fluid-responsive after liver transplant surgery maintained this state. This differs from other ICU contexts, such as sepsis, where fluid responsiveness is inconstant [32,33]. In our patients, the tendency towards early fluid responsiveness post transplantation may suggest that a subset of individuals better adapted from a cardiovascular perspective to significant surgical stress, hence displaying readiness for weaning. These patients can adequately accommodate the intrathoracic positive pressure loss inherent to an SBT and the concurrent increase in venous return, and, owing to their fluid-responsiveness state, respond with an increase in their cardiac index, facilitating an uncomplicated extubation. Conversely, assessing fluid responsiveness by performing a PLR maneuver may serve as a precautionary measure for fluid-unresponsive patients post surgery, signaling a need for fluid removal that can be performed without risking hemodynamic stability [14,34].

It is crucial to recognize that the measured post-surgery fluid balance, often an imprecise estimate [35], could reflect less critical conditions such as fluid redistribution or capillary leak syndrome, which do not necessarily induce cardiac or other organs’ dysfunction. Therefore, our findings support the consideration of fluid responsiveness over immediate fluid balance correction during the weaning process of postoperative liver transplant patients. In any case, the potential benefit of transitioning patients from fluid unresponsiveness to responsiveness before weaning warrants further investigation.

Our insights provide a re-evaluation of clinical practices, advocating for integrating simple, personalized management strategies into standard care protocols. While our findings may contribute to the evidence on PLR utility in post-surgical settings, they are yet to be robustly validated. Our results propose an expanded application of PLR for predicting cardiovascular and respiratory responses to SBTs among pre-weaning surgical patients, an area previously unreported [17].

Our study was limited by its small size, single-center setting, potential assessment bias owing to device technology, unblinded fluid management from the attending physicians, and its observational nature. Therefore, these insights should be interpreted as provisional and call for further research to ascertain the predictive value of PLR tests in broader surgical critical care scenarios.

## 5. Conclusions

Our preliminary observations suggest that, irrespective of their postoperative fluid balance, patients demonstrating fluid responsiveness post liver transplantation may start weaning earlier and achieve successful extubation. This underscores the potential prognostic value of fluid responsiveness as an indicator of sufficient cardiovascular adaptation for weaning in surgical patients and highlights the profound impact of personalized fluid assessment and management on critical surgical care during patient recovery.

## Figures and Tables

**Table 1:** Baseline characteristics of the patients after liver transplantation upon ICU admission.

Variable	Value
Demographics	*n* = 15
Age (years)	62 {54, 65}
Sex (female)	53%
Height (cm)	165 {158, 173}
Weight (kg)	74 {56, 75}
Body mass index	25 {21, 26}
Clinical condition at admission	
APACHE II	14 {10, 18}
MELD	21 {14, 23}
SOFA	6 {6, 10}
Norepinephrine (mcg/kg/min) (6 pts)	0.004 {0.000, 0.220}
Fluid balance (m/L)	4480 {3697, 5723}
Laboratory	
Lactate (mmol/L)	3.2 {1.7, 5.1}
Hemoglobin (g/dL)	9.7 {8.6, 10.5}
Albumin (g/dL)	3.4 {2.7, 4.2}
Na (mEq/L)	142 {138, 143}
K (mEq/L)	4.3 {3.9, 4.4}
BUN (mg/dL)	20 {13, 26}
Creatinine (mg/dL)	0.9 {0.7, 1.2}

APACHE II: Acute Physiology and Chronic Health Disease Classification System II, MELD: model for end-stage liver disease, SOFA: sequential organ failure assessment, BUN: blood urea nitrogen, SBT: spontaneous breathing trial.

**Table 2:** (a) Macrohemodynamic and oxygenation changes during PLR maneuver at ICU admission (T1), PLR maneuver before the SBT (T2), and before and after the spontaneous breathing trial (T3), according to the fluid responsiveness status. (b) Cardiovascular parameter changes during PLR maneuver at ICU admission (T1), PLR maneuver before the SBT (T2), and before and after the spontaneous breathing trial (T3), according to the fluid responsiveness status.

(a)														
FLUID-RESPONSIVE														
	HR (lpm)	*p*	SAP (mmHg)	*p*	DAP (mmHg)	*p*	MAP (mmHg)	*p*	RR (bpm)	*p*	SaO_2_ (%)	*p*	pCO_2_ (mmHg)	*p*
PLR start (T1)	77 {70, 85}	0.971	115 {108, 123}	0.436	47 {47, 60}	0.075	94 {90, 99}	0.280	20 {17, 21}	0.218	100 {99, 100}	0.232		
PLR end (T1)	77 {72, 85}		120 {105, 152}		65 {52, 72}		103 {90, 128}		20 {15, 21}		98 {96, 100}			
PLR start (T2)	79 {69, 88}	0.887	112 {104, 123}	0.551	55 {47, 65}	0.054	92 {85, 104}	0.514	22 {18, 22}	0.143	100 {98, 100}	0.912		
PLR end (T2)	78 {67, 85}		115 {108, 130}		68 {57, 70}		100 {84, 115}		18 {16, 21}		99 {96, 100}			
SBT start (T3)	80 {69, 90}	0.932	114 {104, 132}	0.908	52 {49, 67}	0.219	73 {68, 85}	0.319	20 {19, 22}	0.198	98 {96, 100}	0.413	41 {37, 45}	0.195
SBT end (T3)	75 {70, 91}		116 {103, 130}		62 {53, 70}		78 {73, 88}		18 {14, 21}		97 {96, 98}		37 {33, 39}	
FLUID-UNRESPONSIVE														
	HR (lpm)	*p*	SAP (mmHg)	*p*	DAP (mmHg)	*p*	MAP (mmHg)	*p*	RR (bpm)	*p*	SaO_2_ (%)	*p*	pCO_2_ (mmHg)	*p*
PLR start (T1)	71 {67, 77}	0.527	119 {104, 142}	0.189	64 {59, 66}	0.206	99 {85, 116}	0.401	21 {18, 22}	0.244	99 {98, 100}	0.279		
PLR end (T1)	73 {61, 78}		136 {114, 151}		68 {64, 73}		111 {94, 127}		16 {13, 19}		98 {96, 99}			
PLR start (T2)	76 {72, 76}	0.268	120 {115, 132}	0.050	62 {61, 67}	0.127	105 {99, 116}	0.127	16 {14, 19}	0.658	100 {98, 100}	0.609		
PLR end (T2)	74 {67, 75}		150 {149, 158}		81 {74, 82}		128 {127, 137}		18 {17, 20}		99 {94, 100}			
SBT start (T3)	70 {67, 73}	0.827	123 {119, 146}	0.275	59 {57, 67}	0.121	80 {80, 93}	0.184	16 {14, 19}	0.513	97 {96, 98}	0.822	39 {38, 43}	0.202
SBT end (T3)	71 {66, 74}		149 {148, 165}		77 {75, 77}		106 {105, 112}		15 {11, 18}		97 {97, 98}		37 {33, 39}	
(b)														
FLUID-RESPONSIVE														
		CI (L/min/m^2^)	*p*	SVI (mL/m^2^)		*p*	SVV (%)	*p*	CVP (mmHg)	*p*	SvO_2_ (%)	*p*	TFC (1/Ω)	*p*
PLR start (T1)		2.8 {2.7, 3.0}	0.003	37 {33, 43}		0.005	21 {15, 22}	0.035	9 {8, 10}	0.529	74 {73, 74}	0.971	110 {78, 120}	0.912
PLR end (T1)		4.0 {3.6, 4.7}		55 {49, 55}			13 {13, 16}		9 {8, 10}		73 {68, 75}		113 {75, 116}	
PLR start (T2)		3.1 {2.8, 3.6}	0.005	40 {38, 44}		0.001	16 {14, 21}	0.039	9 {8, 10}	0.681	74 {70, 75}	0.876	94 {72, 115}	0.059
PLR end (T2)		3.9 {3.6, 4.7}		53 {45, 55}			13 {13, 15}		9 {7, 9}		71 {70, 73}		99 {75, 113}	
SBT start (T3)		3.1 {2.8, 3.7}	0.045	42 {32, 48}		0.024	14 {12, 18}	0.713	10 {9, 12}	0.266	72 {70, 75}	0.06	89 {72, 116}	0.755
SBT end (T3)		3.7 {3.4, 4.1}		53 {46, 56}			15 {12, 16}		9 {7, 10}		69 {66, 71}		92 {74, 114}	
FLUID-UNRESPONSIVE														
		CI (L/min/m^2^)	*p*	SVI (mL/m^2^)		*p*	SVV (%)	*p*	CVP (mmHg)	*p*	SvO_2_ (%)	*p*	TFC (1/Ω)	*p*
PLR start (T1)		3.5 {3.1, 4.0}	0.878	47 {43, 51}		0.574	14 {8, 16}	0.721	9 {8, 11}	0.645	71 {69, 74}	0.628	66 {55, 84}	0.994
PLR end (T1)		3.5 {3.1, 4.2}		50 {46, 53}			13 {9, 14}		9 {5, 12}		74 {70, 74}		66 {56, 87}	
PLR start (T2)		3.1 {2.4, 3.4}	0.658	41 {35, 45}		0.513	15 {15, 24}	0.544	9 {8, 10}	0.105	71 {70, 73}	0.784	69 {60, 71}	0.918
PLR end (T2)		3.2 {2.5, 3.5}		43 {36, 47}			13 {12, 23}		8 {7, 9}		70 {69, 72}		69 {58, 72}	
SBT start (T3)		3.2 {2.7, 3.2}	0.268	42 {38, 44}		0.275	16 {12, 19}	0.827	9 {8, 11}	0.993	77 {76, 78}	0.05	51 {50, 58}	0.513
SBT end (T3)		3.5 {2.9, 3.7}		49 {43, 50}			14 {12, 19}		9 {8, 11}		72 {72, 73}		52 {51, 58}	

(a) PLR: passive leg-raising maneuver, SBT: spontaneous breathing trial, HR: heart rate, SAP: systolic blood pressure, DAP: diastolic blood pressure, MAP: mean arterial pressure, RR: respiratory rate, SaO_2_: arterial oxygen saturation, pCO_2_: arterial CO_2_ partial pressure. (b) PLR: passive leg-raising maneuver, SBT: spontaneous breathing trial, SVV: stroke volume variation, CVP: central venous pressure, SvO_2_: central venous oxygen saturation, dCO_2_: venous-to-arterial pCO_2_ difference, TFC: thoracic fluid content.

**Table 3:** (a) Fluid balance in postoperative liver transplant patients according to fluid responsiveness status. (b) Plasma volume status in postoperative liver transplant patients according to fluid responsiveness status.

(a)				
	Fluid balance (postoperative) (mL)	*p*	Fluid balance (before SBT) (mL)	*p*
Fluid-responsive	5230 {3698, 5723}	0.674	4476 {3697, 5722}	0.281
Fluid-unresponsive	4167 {3755, 5583}		2997 {−146, 5747}	
(b)				
	Plasma volume status (postoperative) (%)	*p*	Plasma volume status (before SBT) (%)	*p*
Fluid-responsive	17 {14, 22}	0.156	17 {10, 20}	0.226
Fluid-unresponsive	9 {5, 13}		8 {3, 14}	

## Data Availability

The datasets used and/or analyzed during the current study are available from the corresponding author upon reasonable request.

## Supplementary Materials

The following supporting information can be downloaded at https://www.mdpi.com/article/10.3390/jpm14040429/s1: Table S1. A. Multiple linear regression analysis table showing coefficients examining the influence of some clinically relevant variables on time to spontaneous breathing trial (SBT). B. Multiple linear regression analysis table showing coefficients examining the influence of some clinically relevant variables on time to extubation.
