# Supplementary material for: Fluid Responsiveness Is Associated with Successful Weaning after Liver Transplant Surgery
Source: J Pers Med. 2024 Apr 18;14(4):429. doi: 10.3390/jpm14040429 (PMC11051314; doi:10.3390/jpm14040429)
Supplement: Supplementary file 1 [file jpm-14-00429-s001.zip › jpm-2946254-supplementary.pdf]

Table S1. A. Multiple linear regression analysis table showing coefficients examining the influence of some clinically relevant variables on time to spontaneous breathing trial (SBT). **B**. Multiple linear regression analysis table showing coefficients examining the influence of some clinically relevant variables on time to extubation.

A

	**Unstandardized Coefficients**	**Standardized Coefficients**				**95% confidence interval for B**	
Model	B	Beta	Standard error	t	p	lower bound	upper bound
(Constant)	107.82		62.67	1.72	0.119	-33.94	249.59
FR_state1 fluid responsive	-67.9	-0.77	21.38	-3.18	0.011*	-116.27	-19.53
MELD	-0.05	-0.01	1.48	-0.04	0.972	-3.4	3.29
FB_postop	0	0.06	0	0.29	0.781	-0.01	0.01
CI	-43.94	-0.78	14.76	-2.98	0.016*	-77.34	-10.55

* Statistically significant

FR_state fluid responsive positive fluid responsiveness state at T1, *MELD* model for end-stage liver disease, *FB_postop* postoperative fluid balance, *CI* cardiac index.

The regression model showed that the overall fit was statistically significant (p = 0.026). Being fluid responsive and having a higher cardiac index are significantly associated with a shorter time to SBT this sample of liver transplant patients with high perioperative fluid balance.

B

	**Unstandardized Coefficients**	**Standardized Coefficients**				**95% confidence interval for B**	
Model	B	Beta	Standard error	t	p	lower bound	upper bound
(Constant)	107.06		64.3	1.66	0.130	-38.39	252.51
FR_state2 fluid responsive	-69.22	-0.8	21.94	-3.16	0.012*	-118.85	-19.59
MELD	0.18	0.03	1.52	0.12	0.909	-3.25	3.61
FB_postop	0	0.06	0	0.28	0.788	-0.01	0.01
CI	-41.22	-0.74	15.15	-2.72	0.024*	-75.49	-6.96

* Statistically significant

FR_state2 fluid responsive positive fluid responsiveness state at T2, *MELD* model for end-stage liver disease, *FB_postop* postoperative fluid balance, *CI* cardiac index.

The regression model showed that the overall fit was statistically significant (p = 0.041). Being fluid responsive and having a higher cardiac index are significantly associated with a shorter time to extubation in this sample of liver transplant patients with high perioperative fluid balance.
